# The efficacy of social skills training in the treatment of schizophrenia

**DOI:** 10.1192/j.eurpsy.2021.1351

**Published:** 2021-08-13

**Authors:** F. Brando, G.M. Giordano, G. Piegari, D. Palumbo, P. Bucci, A. Mucci, S. Galderisi

**Affiliations:** Department Of Psychiatry, University of Campania “Luigi Vanvitelli”, Naples, Italy

**Keywords:** social skills training, schizophrénia, psychiatric rehabilitation, social cognition

## Abstract

**Introduction:**

Social cognition and skill deficits have been largely documented in subjects with schizophrenia (SCZs), and have a strong influence on the functional outcome of these subjects. Different behavioural interventions have been developed to target and improve social skills in SCZs. For instance, the Social Skills Training (SST) focuses on improving communication skills and assertiveness to facilitate disease management, independent living and real-life functioning of SCZs. SST seems also to have an impact on negative symptoms and social cognition.

**Objectives:**

The study aims to evaluate the effectiveness of SST in improving social cognition and negative symptoms in SCZs.

**Methods:**

The sample included 8 chronic SCZs (age between 18 and 60), who completed 6 months of SST. The intervention consisted of two weekly group sessions of 2 hours each. We assessed psychopathology, neurocognition, real-life functioning, functional capacity and social cognition at baseline and after training. Paired samples t-tests were performed to evaluate the differences of the variables considered after completing the treatment.

**Results:**

Significant improvements in negative symptoms (p<.05), social cognition (p<.05), functional capacity (p<.001), activities of daily living (p<.001) and interpersonal relationships (p<.011) were found.

**Conclusions:**

The present findings suggest that SST might ameliorate social cognition and negative symptoms which are generally not influenced by antipsychotic treatment. The integration of pharmacological and SST interventions might have an impact on major determinants of poor real-life functioning in SCZs.

